# The Brain Injury Screening Tool (BIST): Tool development, factor structure and validity

**DOI:** 10.1371/journal.pone.0246512

**Published:** 2021-02-04

**Authors:** Alice Theadom, Natalie Hardaker, Charlotte Bray, Richard Siegert, Kevin Henshall, Katherine Forch, Kris Fernando, Doug King, Mark Fulcher, Sam Jewell, Nusratnaaz Shaikh, Renata Bastos Gottgtroy, Patria Hume

**Affiliations:** 1 TBI Network, Auckland University of Technology, Auckland, New Zealand; 2 School of Clinical Sciences, Auckland University of Technology, Auckland, New Zealand; 3 Accident Compensation Corporation, Wellington, New Zealand; 4 Sports Performance Research Institute New Zealand, Auckland University of Technology, Auckland, New Zealand; 5 Trauma Service, Counties Manukau District Health Board, Auckland, New Zealand; 6 Axis Sports Medicine, Auckland, New Zealand; 7 Active+, Auckland, New Zealand; 8 School of Science and Technology, University of New England, Armidale, New South Wales, Australia; 9 Wellington Sports Med, Wellington, New Zealand; University of Florida, UNITED STATES

## Abstract

Currently health care pathways (the combination and order of services that a patient receives to manage their injury) following a mild traumatic brain injury vary considerably. Some clinicians lack confidence in injury recognition, management and knowing when to refer. A clinical expert group developed the Brain Injury Screening Tool (BIST) to provide guidance on health care pathways based on clinical indicators of poor recovery. The tool aims to facilitate access to specialist services (if required) to improve longer term prognosis. The tool was developed using a three-step process including: 1) domain mapping; 2) item development and 3) item testing and review. An online retrospective survey of 114 adults (>16 years) who had experienced a mild brain injury in the past 10 years was used to determine the initial psychometric properties of the 15-item symptom scale of the BIST. Participants were randomised to complete the BIST and one of two existing symptom scales; the Rivermead Post-concussion Symptom Questionnaire (RPQ) or the Sports Concussion Assessment Test (SCAT-5) symptom scale to determine concurrent validity. Participant responses to the BIST symptom scale items were used to determine scale reliability using Cronbach’s alpha. A principal components analysis explored the underlying factor structure. Spearman’s correlation coefficients determined concurrent validity with the RPQ and SCAT-5 symptom scales. The 15 items were found to require a reading age of 6–8 years old using readability statistics. High concurrent validity was shown against the RPQ (*r* = 0.91) and SCAT-5 (*r* = 0.90). The BIST total symptom scale (α = 0.94) and the three factors identified demonstrated excellent internal consistency: physical/emotional (α = 0.90), cognitive (α = 0.92) and vestibular-ocular (α = 0.80). This study provides evidence to support the utility, internal consistency, factor structure and concurrent validity of the BIST. Further research is warranted to determine the utility of the BIST scoring criteria and responsiveness to change in patients.

## Introduction

Traumatic brain injury occurs where there is an external force causing an alteration in brain function [1]. One of the critical acute decisions clinicians need to make, is to determine the risk of bleeding and swelling in the brain following injury. This includes establishing if any of a range of clinical indicators for further diagnostic imaging are present. Clinical indicators include repeated vomiting, worsening symptoms, age over 65 years, suspicion of skull fracture, severe headache, more than brief loss of consciousness, use of coagulation impacting therapies and post-injury seizure [2].

Many people recover well in the days to weeks following mild traumatic brain injury (mTBI), however, over 40% can experience persistent symptoms that impact on daily living for many years [3–5]. Research has shown that risk factors for poor recovery outcome include a history of (previous) TBI, a pre-existing mental health condition, delayed medical attention (including patient delays in seeking treatment), older age, maladaptive coping, and increased severity of acute symptoms [6, 7]. There is increasing evidence that early identification and good recovery advice improves longer term recovery [8–10]. However, currently access to specialist services requires the patient to know to go back to their General Practitioner (GP) if they do not recover spontaneously. Further the GP needs to be aware of available services and processes to support a patient’s recovery. The current pathway can create significant barriers or delays in treatment.

Given up to 95% of TBIs are classified as being mild in severity, many injuries can be managed outside of the hospital context. However, currently treatment can vary widely both within and between countries and can often depend on the experience and expertise of the treating clinician on initial medical presentation [11]. There are a number of clinical indicators and risk factors for clinicians to consider as part of decision making on the appropriate health care pathway for a patient. It can be challenging for clinicians to be aware of all the specific indicators particularly if a clinician is less experienced or does not see many mTBI’s within their clinical practice. Indeed, studies have shown that many primary care practitioners lack confidence in recognising and managing mTBI effectively and knowing when to make referrals to specialist services [12]. There are also increasing calls to support other health professionals such as physiotherapists, occupational therapists and nurse practitioners who frequently assist in recognition of possible mTBI’s [13].

Tools designed to try and subclassify mTBI’s based on injury type, level of consciousness and alterations in mental state post-injury have not been found to adequately discriminate mTBI’s or to predict how a person will recover [14]. Consequently, the acute assessment now focuses on a more comprehensive assessment of acute symptoms such as the severity of headaches, dizziness and disturbed vision. Two of the most commonly utilised symptom scales include the Sports Concussion Assessment tool (SCAT 5) [15] and the Rivermead Post Concussion Scale (RPQ) [16]. The SCAT-5 was designed specifically for the sports context to assist in concussion recognition and diagnosis. For example, the memory questions ask ball sport-specific questions such as “which half is it now” which need to be adapted for different sports. The tool is widely used across different sports across the globe and has continued to be refined over five versions to improve clinical utility. However, there is less evidence for applicability of use outside of the sports context. An international review revealed that 40/67 (60%) countries have primary care consultations with an average duration of 10 minutes or less [17]. Yet it is acknowledged that the SCAT-5 cannot be performed correctly in less than 10 minutes [15] making its utility within a primary care context challenging. The psychometric properties of the SCAT-5 have also been found to be poor [18] and its authors have acknowledged its limited role in tracking recovery and assisting the return to play/sport decision [15]. Additionally, there is little support for the SCAT-5 as a unidimensional measure of severity [19]. Symptoms scales, such as the RPQ [16] were designed for the research context to assist in determining the type and frequency of symptoms experienced following a TBI. However, the underlying factor structure of the RPQ has been found to vary considerably making it difficult to use total or subscale scores in outcome prediction and it has limited utility on clinical pathway decision making [19, 20].

There is also evidence that dominant symptom clusters, such as high physiological symptom reporting, may be additional predictors of prolonged recovery [21]. Particular symptom clusters may also indicate where specific professional input is needed early in treatment (e.g. a referral for neurophysiotherapy) [21–23]. Use of different tools and concussion policies across different situational contexts and sports is confusing and inefficient for clinicians and patients alike increasing variation in health care delivery. Consequently, there is a need for consistent, equitable access to concussion recognition and best practice management.

The aims of this study were to develop and validate a short, brain injury screening tool for use at initial assessment that could be utilised:

- across a diverse an age range;
- for injuries sustained through many situational contexts (e.g. sport, school, work, violence or everyday living activities);
- across varied health care settings (e.g. general practice, urgent care or Accident and Medical Setting, allied health clinics, physiotherapy);
- without the need for specialist training; and
- to provide guidance to clinicians on initial health care pathways.

## Materials and methods

A clinical expert group (CEG) was established comprised of general practitioners, sport and exercise medicine physicians, physiotherapists, trauma nurses, psychologists, TBI researchers and service funding representatives. A three step process was undertaken to develop the BIST drawing on the methods used by Seela et al [24] and outlined in Table 1.

**Table 1:** Measure development process.

Step 1—Domain mapping	Step 2—Item development	Step 3—Item testing and review
Define context for use and potential operational constraints Quick to administer To be able to be used without need for specialist training For use across range of clinical settings and injury contexts Three potential initial health care pathways	Review items within existing symptom scales	Initial review of the tool by practitioners across a range of clinical contexts to determine clinical utility
Define population to be targeted Those with suspected brain injury aged over 8 years	Identify current clinical guidelines	Feedback from patients on wording of symptom items
Review literature to identify predictors of recovery	Generation of initial item pool based on existing scales and clinical guidelines	Review of missing data that may indicate problematic items
Review advantages and disadvantages with existing tools	Clinical expert group discussion into items required into key items needed in acute phase for initial health pathway decision making	Determine concurrent validity with two existing tools.
Identify potential risks Recognition tool not a diagnostic tool Encourage administration in conjunction with other tests as determined by clinician	Review of item wording	Determine internal consistency and factor structure
Establish scope of tool to be designed	Develop conceptual framework of clinical subscales	Modify and review items based on findings
	Meeting with service providers to determine scoring cut offs with clinical relevance	

Following the CEG’s review of the existing literature and assessment tools against the identified needs and purpose (Table 1), a number of key limitations were identified. Current symptom scales often include complex terms (e.g. nausea and noise sensitivity) that may make accurate reporting for patients challenging. This is a particular issue for people whose first language is not English. Existing tools also do not support clinical decision making as to how the patient should best be managed across all health care pathways. For example, determining whether a patient should be referred to the emergency department, refer for early specialist treatment (if they are at risk of long-term problems) and those who can be monitored and managed in primary care. The time taken to administer some tools such as the SCAT-5 was also highlighted as a limitation on search for a measure for use. Based on the identified constraints the CEG determined the need for development of a new symptom measure called the Brain Injury Screening Tool (BIST).

To develop the BIST, the CEG debated the most important symptoms that would need to be considered clinically at the initial stage to determine care pathways for the patient e.g. to determine their level of risk of acute complications/sequelae (high risk patients requiring urgent hospital care), those at risk of longer-term problems (medium risk) needing early support and those likely to recover with monitoring and initial advice (low risk). One of the operational considerations identified was that as many symptoms cannot be observed until at least 24 hours after an injury (e.g. sleep quality), these symptoms should be made optional depending on the time since injury that a person presents for medical treatment. There were 11 items identified by the CEG that could be assessed within 24 hours of injury and an additional four items that could be assessed if the person presented 24 hours post-injury. The additional items were included to allow a more comprehensive symptom assessment and to monitor recovery. The draft BIST was taken out for consultation with practitioners outside of the CEG working in clinical settings, to obtain initial feedback and to identify and operational barriers to potential implementation. Feedback included the need to ensure that national criteria for neuroimaging and hospital review was captured by the tool, which was subsequently undertaken, and additional screening questions added. The final tool is available from https://tbin.aut.ac.nz/.

### Procedure for the validity study

To determine initial validity of the tool a cross sectional study of people following a mild TBI was completed. Permission to conduct this study was obtained from the Auckland University of Technology Ethics Committee (AUTEC reference number– 20/121). Adults aged over 16 years, who had experienced at least one TBI in the past 10 years were recruited via social media, concussion clinics and sports organisations over a 4-month period between May and September 2020. Adults interested in taking part were able to access a weblink that took them to a Qualtrics website that provided information about the study and what would be involved. Participants were asked to read the consent form and then if they wished to take part to indicate their consent in the study by ticking a box agreeing to take part in the study (online consent). The participants were then able to proceed with answering the questionnaire online including sociodemographic characteristics and about their brain injury history. They were then randomised within the Qualtrics survey to receive one of the following combinations of assessments; the BIST and the symptom scale of the SCAT-5, or the BIST and the RPQ to determine concurrent validity. At the end of the survey, participants were asked to comment on how they found completing the BIST. Those who completed the questionnaire were then invited to enter a prize draw for one of three $100 fuel vouchers. If interested, participants were taken to a separate questionnaire to enter in their contact details to protect their confidentiality, which was drawn following completion of data completion. Contact details were then deleted.

### Measures

The BIST consists of two components. The first component comprises of nine questions used to determine if a patient is at ‘high risk’ via a description of what happened and specific questions aiming to identify any ‘red flags’ or clinical indicators suggesting that the person may need an urgent referral to hospital (e.g. repeated vomiting). The second component includes a 15-item symptom report scale. People are asked to rate how much they now experience the symptoms listed on a scale of 0 (not at all) to 3 (severe). The BIST was initially designed for those aged 8 years and over and to have a clinical conceptual framework of five subscales: physical, vestibular, cognitive, emotional and sleep.

The SCAT-5 [15] was designed to support the assessment of suspected concussion within the sports context in those aged over 13 years. A child version of the SCAT-5 is available for those aged 5–12 years old. It can also be used as a baseline measure with athletes to enable comparison with post-injury responses. The SCAT-5 consists of a short relevant medical history, observable signs, a cognitive and neurological screen, and symptom evaluation and is repeated over time to monitor recovery in sport to support diagnosis of concussion. It can only be administered by a trained healthcare professional. The symptom scale consists of 22 symptoms rated on a scale of 0 (none) to 6 (severe). The SCAT-5 has demonstrated high test retest reliability [25] and adequate internal consistency [26].

The RPQ [16] assesses the presence and severity of 16 symptoms commonly experienced following a brain injury compared to pre-injury. Participants rate each symptom using a five-point scale ranging from 0 (absent) to 4 (severe problem). The RPQ was designed for adults following mild to moderate brain injury aged 18 years plus. The RPQ has demonstrated excellent test re-test reliability [16] and internal consistency [27].

### Statistical analysis

Individual participant feedback comments were explored, and any suggested modifications identified and listed. Frequencies were used to determine proportion of missing data on each of the 15 symptom items. Scale reliability was assessed using Cronbach’s alpha (α) with a value of 0.70 considered the minimally acceptable level of reliability. The number of factors underpinning the BIST were determined by a scree plot and principal components exploratory factor analysis. As the factors were expected to be correlated, a direct oblimin rotation was used that only included items with eigenvalues >1. Spearman’s correlation coefficients were used to determine concurrent validity between responses on the BIST and the RPQ and SCAT-5.

## Results

Of the 163 adults who consented to take part in the study, 118 (72.4%) completed the questionnaire. Data were then checked against the inclusion criteria to determine eligibility; four participants were excluded as the date of the last TBI was >10 years ago. Data were analysed for the final sample of 114 participants. A minimum of 75 participants were required to ensure sufficient sample size for this analysis (e.g. a minimum of five people per item of the BIST—which consisted of 15 items) [28]. Average time to complete the full questionnaire (including both the BIST and one other measure) was 7.4 (± 115.5) minutes.

Participants ranged in age between 16 and 72 years, with a mean age of 32.4 years (± 13.6). The average time since last TBI was 2.1 years (± 2.29). Table 2 summarises the characteristics of the participants included in the analysis.

**Table 2:** Participant characteristics for the sample of N = 114.

	N (%)
**Sex**	
Male	23 (20.2)
Female	90 (78.9)
Missing	1 (<1%)
**Ethnicity**	
European	100 (87.7)
Non-European	14 (12.3)
**In a relationship**	
Yes	75 (65.8)
No	36 (31.6)
Missing	3 (2.6)
**Highest level of Education**	
Secondary school	20 (17.5)
College/Professional training	14 (12.3)
University	78 (68.4)
Missing	2 (1.8)
**Number of TBIs sustained over lifetime**	
1	42 (36.8)
2–3	39 (34.2)
4–5	18 (15.8)
6–7	10 (8.8)
8+	4 (3.5)
Missing	1 (0.9)
**Cause of injury for last TBI**	
Accidentally hit by an object/person/animal	49 (43.0)
Assault	10 (8.8)
Fall	37 (32.4)
Traffic accident	14 (12.3)
Other	2 (1.8)
Missing	2 (1.8)
**Context of injury for last TBI**	
Activity of daily life	17 (14.9)
Travelling	14 (12.3)
Sport	68 (59.6)
Work	5 (4.4)
Other	9 (7.9)

### Usability of the BIST

There were only five items (33%) for which at least one data point was missing. There were no specific questions identified with high levels of missing data indicating a problem with that item. Only one or two people omitted data on these five items and the data were therefore deemed to be missing at random. Feedback from adult participants supported the readability and applicability of the items with comments including “easy for people to understand”, “very easy to follow”, “easy to complete” “nice and simple”, and “good questions that felt relevant”.

One clarification was highlighted by participants about whether they should report their symptom experience for right now (at this point in time), when the injury happened or when symptoms were at their worst? The instructions on the tool were amended from “Compared to before your accident, do you have any of the following?” to “Compared with before the accident, please rate how much you experience the following right now (at this point in time)”. One participant highlighted that changes in her emotional reactions were not captured by the existing item focusing on being annoyed “my main issue is that I anger quicker and have less control over my emotions.” Discussion of this item with clinicians also highlighted that term “annoyed” may be too broad and could reflect a number of issues not necessarily related to the injury. The item “I get easily annoyed” was changed to “I get angry quicker”.

Discussions with clinicians and service providers on the 0–3 response scale indicated that a 0–10 scale would be better due to it being in line with clinical advice which advocates monitoring of symptoms on a 0–10 scale and to enable smaller changes in symptoms to be detected over time. It was also suggested there was a need to be clearer about which criteria put patients as high, medium, or low risk. An additional question on the impact of symptoms on the person and their life was also proposed as a more global measure of impact, and as symptoms do not always correlate with the impact on a person’s life.

### Readability statistics

Based on a combination of seven readability statistics (including a Flesch Reading Ease score = 96.5, Gunning Fog = 3.7, Flesch-Kincaid Grade Level: 1.2 First Grade, The Coleman-Liau Index: 5, Fifth Grade, The SMOG Index: Third Grade, Automated Readability Index: -0.4, 3–5 yrs. old (Preschool) and Linsear Write Formula: 2, Second Grade) the 15 BIST items were classified overall as ‘very easy to read” with a reading age of 6–8 years old [29].

### Internal consistency

Total scores on the BIST symptom scale ranged between 0–44, with a median score of 15 (interquartile range of 15.75). The Cronbach’s alpha for the 15-item symptom scale of the BIST was 0.94, indicating excellent scale reliability. This suggests that the 15-items of the BIST are closely related and are measuring the same construct. This compares favourably with previous evidence for both the RPQ (0.95) [27] and the SCAT (0.94) [18].

### Underlying factor structure of the BIST

**Fig 1:**
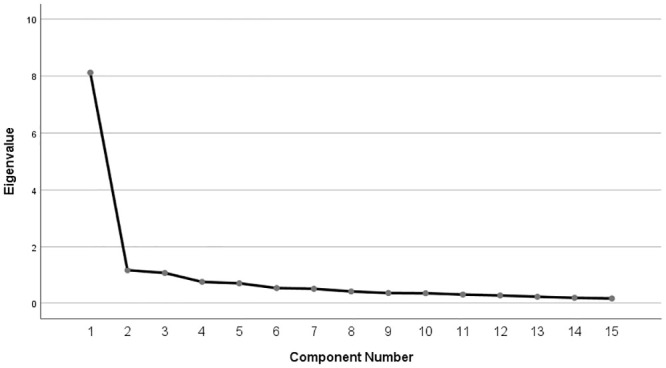
Scree plot for principal components analysis of the BIST.

The data were found to be suitable for factor analysis with a Kaiser-Meyer-Olkin measure of sampling adequacy of 0.88. As shown in Fig 1, the scree plot indicated a two or three factor structure of the BIST.

Looking at the output from the principal component analysis, an item was assumed to load on a given factor if the factor loading was at least 0.40. There was moderate correlation between the items supporting a link to the same underlying construct but limited overlap. There were no items identified as not contributing significantly to the overall measure as shown in Table 3.

**Table 3:** Relationships between items within the BIST and contribution of items to the overall scale.

	Corrected Item-Total Correlation	Cronbach’s Alpha if Item Deleted
I have trouble concentrating	0.822	0.938
I get confused easily	0.803	0.938
I feel tired during the day	0.799	0.938
It takes me longer to think	0.765	0.939
I forget things	0.743	0.940
I don’t like bright lights	0.737	0.940
I am easily annoyed	0.731	0.940
I don’t like loud noises	0.720	0.940
I sleep a lot more or can’t fall asleep	0.714	0.941
If I close my eyes, I feel like I am at sea	0.695	0.941
I have trouble with my eyesight (vision)	0.628	0.942
Headaches (my head hurts)	0.617	0.942
I feel restless	0.674	0.941
My neck hurts	0.574	0.943
I feel like I will be sick	0.565	0.944

As shown in Table 4, the proposed component structure of the BIST based on clinical relevance was well supported. The relevant items in the proposed vestibular-ocular and cognitive components strongly loaded on to the equivalent factors. The items in the physical and emotional and sleep components were found to load onto the same single factor (renamed physical and emotional). Loadings below 0.30 were removed from the table.

**Table 4:** Item loadings on the three factors of the BIST.

	Rotated Factors		
	Physical and Emotional	Cognitive	Vestibular-ocular
Headaches (my head hurts)	0.519		
My neck hurts.	0.528		0.490
Don’t like bright lights.	0.551		
Don’t like loud noises.	0.685		
I am easily annoyed.	0.519		
I feel restless.	0.461	-0.313	
I feel tired during the day.	0.873		
I sleep a lot more or can’t fall asleep.	0.948		
If I close my eyes I feel like I am at sea.			0.715
I feel like I will be sick.			0.876
I have trouble with my eyesight (vision).			0.747
It takes me longer to think.		-0.726	
I forget things.		-0.921	
I get confused easily.		-0.810	
I have trouble concentrating.		-0.833	

Highlighted boxes indicate the factor each item has highest loading onto.

A reliability index was calculated to assess the internal consistency of the identified three factors. The scale reliability of each of the components/factors was supported, with a Cronbach’s alpha of α = 0.90 for the physical and emotional component, α = 0.92 for the cognitive component and α = 0.80 for the vestibular-ocular component. The lower alpha of the vestibular factor can be attributed in part to the small number of items within this subscale. There were moderate correlations between the three factors physical/emotional: cognitive *r* = 0.71, physical/emotional:vestibular *r* = 0.69, cognitive:vestibular *r* = 0.64.

### Concurrent validity

As the data were moderately skewed a Spearman correlation co-efficient was used. There was a very strong correlation between the BIST and the RPQ (*r* = 0.91) and between the BIST and the SCAT-5 (*r* = 0.90) supporting excellent concurrent validity with existing measures.

## Discussion

The findings of this study provide initial support for readability, scale reliability and use of the BIST as a total symptom scale and subscales. However, some modifications were highlighted including changes in wording from “I get annoyed” to “I get angry or irritated more easily”. The response scale was also changed from 0–3 to 0–10, clarifying the instructions and including an overall impact rating.

Time to complete the BIST and readability of the symptom items support the potential utility of the BIST as an initial assessment tool that could be used within busy clinical environments to support consistency of care. There was support that patients could also easily self-complete the symptom scale (second component of the BIST). This finding supports the potential for the symptom scale to be used by patients to monitor their symptoms over time and support the identification of symptom exacerbation triggers. Automation of the BIST could assist clinicians with scoring of the measure and facilitate the symptom monitoring process through enabling patients to complete the symptom items over time and providing graphs to track the recovery journey. This would assist clinicians in determining whether a patient’s symptoms had plateaued or were deteriorating indicating a need for specialist treatment and for patients to see progress even if it may be small. There is evidence that having a way to monitor progress would assist patients in their recovery journey [30]. Automation of the tool may also allow for large amounts of data to be collected that is easily accessible, enabling novel machine learning and statistical methods to be applied to build predictive models to aid in cut off scores for further refinement of care pathways and the factors affecting prognosis.

The excellent scale reliability of the total scale and the three factors of the BIST supports its potential use in adults exploring the predictive value of symptom clusters on recovery. There is increasing evidence that symptom clusters may be useful in identifying people who may need early additional support and specifically the type of support they need [21–23]. Evidence for use of the BIST total score will assist in determining a cut off score that may help distinguish people needing early specialist intervention and those who can be given early advice and monitored in primary care to assist with resource allocation, particularly given a high proportion of people do recover naturally [4, 5].

### Limitations of this study

To determine initial feasibility and validity of the scale and to enable comparison with other measures, only adults were included in this analysis. Specific studies are needed to determine utility of the measure for those aged 8–15 years. There appeared to be a lower proportion of males within the current analysis than would be expected in the mTBI population, where males are at higher risk of mTBI. Most participants in the current analysis were several years post-injury and had a good level of education. Piloting of the measure within an acute setting is now warranted. Responsiveness, predictive validity, and test-retest reliability were unable to be explored within this study.

## Supporting information

S1 File (PDF)
